# Exploring the Impact of the 2020 COVID‐19 Adjusted Lockdown on Social Workers at a Gender‐Based Violence Command Centre in Pretoria, Gauteng

**DOI:** 10.1002/puh2.70137

**Published:** 2025-10-07

**Authors:** Qebo Ignatia Nkosi, Leane Ramsoomar

**Affiliations:** ^1^ School of Public Health and Health Systems University of Pretoria Gauteng South Africa; ^2^ Gender and Health Research Unit South African Medical Research Council Pretoria South Africa

**Keywords:** COVID‐19, GBV, social worker

## Abstract

**Introduction:**

In South Africa and across many regions globally, adjusted lockdowns were implemented as a measure to curb the spread of COVID‐19. These lockdowns had a significant effect on gender‐based violence (GBV). Victims, often confined with their abusers, faced limited access to support services. Simultaneously, healthcare workers, including those at the Gender‐Based Violence Command Centre (GBVCC), were at the forefront of the pandemic response, dealing with both COVID‐19 and GBV cases.

**Method:**

A phenomenological approach was used to collect data from nine social workers at the GBVCC in South Africa. Semistructured interviews were conducted, guided by an interview protocol. Data were analyzed using Tesch's framework for thematic analysis.

**Results:**

The findings revealed that social workers faced mental health challenges, including anxiety, stress, and fear of death, during the adjusted lockdown. Many relied on family and friends for emotional support. The work environment was notably challenging, and this, in turn, affected their job performance.

**Conclusion:**

The adjusted lockdown had a profound impact on social workers at the GBVCC, affecting both their personal well‐being and professional responsibilities. Many reported experiencing poor mental health and a disruption in work–life balance during this period.

## Graphical abstract

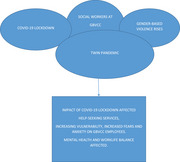

## 1 Introduction and Literature Review

The COVID‐19 pandemic exacerbated the existing crisis of gender‐based violence (GBV) in South Africa, reflecting a broader trend seen globally. The conditions of lockdowns, intended to stem the spread of the virus, inadvertently contributed to an increase in GBV, a phenomenon often referred to as a “shadow pandemic” [1]. This increase was propelled by factors such as economic stresses, confinement, and isolation, which heightened tensions and provided opportunities for abusers within households.

In South Africa, a country already grappling with high levels of intimate partner violence (IPV) and domestic abuse, the lockdown measures had particularly severe implications. A study focusing on women's experiences during the South African lockdown found that the public health measures not only increased women's exposure to IPV but also limited their access to support services and escape routes [2]. The escalation of violence under lockdown conditions highlighted the gender‐insensitive aspects of pandemic control measures, indicating that stay‐at‐home orders and travel restrictions likely magnified the risks for women trapped in abusive situations.

Preliminary reports from South African police and other services suggest that GBV cases were on the rise during the lockdown, with women and children being the most affected groups. One study showed that GBV had increased in South Africa, especially during COVID‐19 [3]. The research conducted outside of South Africa found that during COVID‐19, there was a general increase in violations of women's rights [4]. In Uganda, there were cases of GBV in the presence of decreased capacity to support victims on the part of the justice sector [3]. The study found that most services were affected by the lockdown, including essential services such as police stations, which were often out of reach during crucial times. Victims of GBV suffered at the hands of their perpetrators, and the justice system was also slow at responding to emergencies.

Research conducted in the Gauteng province of South Africa highlighted how the lockdown negatively impacted families, exacerbating risk factors for domestic violence. Economic pressures such as job losses and food insecurity were significant drivers of violence in low socioeconomic families. In contrast, the confinement with abusive partners in more affluent families intensified relational conflicts and violence [5]. The heightened economic stress and lack of psychosocial support in those families during this period further contributed to the strain on domestic environments, which was a common theme across various socioeconomic groups.

Among refugee women in Durban, South Africa, the pandemic intensified existing insecurities and structural violence. Refugee women, often marginalized in society, faced exclusion from targeted COVID‐19 support measures, thereby amplifying their vulnerability [6]. This context of marginalization exacerbated the impact of the pandemic on their well‐being, demonstrating the interconnectedness of public health crises and social vulnerabilities.

As a national response strategy for GBV, social workers, employed by the Department of Social Development to render psychosocial services in the Gender Based Violence Command Centre (GBVCC), were called upon to continue working 12‐hour shifts as per the original conditions of employment, which included day and night shifts to respond to GBV on a call‐center basis. The healthcare response to the COVID‐19 pandemic led to these professionals observing double pandemics. Responding to any pandemic depends upon a mentally and physically healthy workforce; however, a systematic review on the COVID‐19 pandemic and mental health [7] and a narrative review on COVID‐19‐related mental health effects in the workplace [8] both concluded that COVID‐19 resulted in increased levels of depression, anxiety, and poor sleep quality. Given the magnitude and impact of the COVID‐19 pandemic and the burden of GBV in South Africa, at every stage of the pandemic, it is necessary to take into account people's psychological and psychiatric needs [9]. Despite several new studies on the impact of COVID‐19 on other frontline workers, including doctors, nurses, police officers, and teachers, little is known about the experiences of social workers who were serving victims and survivors of GBV during the COVID‐19 adjusted lockdown [10, 11]. Almost five years into the pandemic, there is value in reflecting on experiences and lessons learnt from employees at the front‐line of the pandemic response in the GBVCC.

## 2 Research Methods

The study was conducted using a qualitative approach and employed a phenomenological design based on studying individuals’ lived experiences. The study sample included a total of 9 social workers (6 females and 3 males), who participated voluntarily in interviews, which were conducted in English, in keeping with the predominantly spoken language in the center. All social workers who participated were employed as public servants at the GBVCC. These participants were purposively selected based on fitting the inclusion criteria: (1) Social workers who were employed at the GBVCC in Pretoria, who were on duty throughout the COVID‐19 adjusted lockdown. (2) All social workers, regardless of demographic factors such as age, race, and gender, who were employed at GBVCC during the COVID‐19 adjusted lockdown. (3) All social workers who worked a 12 h shift in the GBVCC during the COVID‐19 adjusted lockdown.

## 3 Data Collection and Analysis

A trained research assistant initiated data collection by asking social workers to read an information sheet, ask questions, and sign a consent form in a private setting. Participation was voluntary, with the option to withdraw at any time without consequences. In‐depth interviews were conducted using a semistructured guide, with each session lasting for 60–80 min.

The study data were analyzed using Tesch's method of analysis [12]. The Tesch method is a framework that involves eight steps for analyzing qualitative data. Some steps in data analysis involved listening to audiotaped interviews and reading verbatim transcripts [13]. Similar information was coded and categorized as key themes and supporting subthemes [14]. Data and themes were analyzed by the first author, and then discussed and verified with the second author.

## 4 Data Trustworthiness

Trustworthiness was ensured through credibility, dependability, transferability, and confirmability [15]. Credibility involved minimizing bias by using a trained public health practitioner for data collection and carefully selecting questions to reduce recall bias. Dependability was achieved through data saturation, reached between participants 7 and 9 [14], and by maintaining flexibility in the semistructured interviews. Transferability was supported by referencing existing literature and providing detailed descriptions of the research context. Confirmability was addressed by recording participants’ contact details to validate findings against their experiences.

## 5 Ethical Considerations

This research study was conducted in keeping with the principles of the Helsinki Declaration, which involves respect for personal autonomy, justice, beneficence, and nonmaleficence when conducting research involving human subjects [16]. Given the sensitivity of the research topic, the researcher organized services for psychosocial support for social workers who may experience trauma as a result of the study. Ethical approval for the study was obtained from the University of Pretoria Research Ethics Committee, and permission was granted by the National Department of Social Development to utilize GBVCC premises. Consent forms were completed by all participants.

## 6 Results

### 6.1 Emotional and Mental Health Experiences

#### 6.1.1 Anxiety and Depression

A major theme emerged from the findings related to anxiety and depression, frequently recounted by many participants. Anxiety was reported as a consistent experience among all the social workers during the lockdown period. They referred to the Level 5 hard lockdown (when the most stringent conditions were imposed) as the most difficult period in which they had to adjust immediately to restrictions and the uncertainty brought about by the COVID pandemic.

“The fear, the anxiety, and all. I wouldn't really say stress went down as the levels were adjusted, but yeah, I don't know how to put it, but I wouldn't really say it reduced the level of stress or anxiety; I think we just adapted” (P8, 40 female).

#### 6.1.2 Fear of Death

Fear of death emerged overwhelmingly in almost all participant accounts, as social workers reported that they feared for their lives and that of their loved ones. Most felt that lockdown level 4 had a greater toll of deaths than other levels. This heightened their fear of death and the fear associated with the mounting deaths of others, considering that these participants were also observing GBV at the time of this experience.

“Joh, when I hear about the people that lost their lives, that was overwhelming, it was terrifying, yeah, when we heard about the statistics that are going up and the number of dying, definitely it was overwhelming” (P1, 38 female).

“I think level 5 didn't have much of the reported deaths, I think level 4 was the hardest because now the numbers were increasing and the people that we knew were dying, so I think level 4 was the hardest” (P7, 40 male).

When asked what was the most difficult experience for them during the lockdown period, one participant responded:

“It has to be the thoughts of dying or losing someone to COVID. At some point, I changed my will, you know, COVID taught us things. Now, when I think about it, I feel like at some point we were preparing for our own funerals. When I had COVID, I expected to die at any time, and that was the scariest moment of my life, especially when I thought of my children” (P5, 16 female).

### 6.2 Coping Mechanisms

#### 6.2.1 Family Institution as a Mechanism

In the face of anxiety, stress, and fear, most social workers found that talking to family members, friends, and colleagues helped them cope with all levels of lockdown.

“I think for me, what happened during lockdown, I had family members so they were a source of support, friends, and I also had my colleagues that we would talk about these things and would encourage one another, yeah” (P3, 34 male).

#### 6.2.2 Coping Behaviors

In the face of stress, anxiety, and fear, participants explained how they engaged in certain coping behaviors, such as overeating, while also spending more time with family, while others relied on self‐talk, patience, and understanding that the pandemic period would pass. This indicated a positive side when some participants indicated that family institution had a supportive impact on their coping strategy.

“Normally, when I am stressed, I eat a lot, so during COVID, I was eating every chance I got. The weight I am struggling to lose is all from that time. I think that is what I can say caused me depression. Otherwise, I was not feeling alone or lonely as such because family was always there” (P6, 20 female).

#### 6.2.3 Debriefing Sessions by GBVCC

The Department of Social Development arranged debriefing sessions in which social workers were allocated psychologists from an external company to provide psychosocial support. Interestingly and perhaps surprisingly, some social workers stated that their information was not handled confidentially by psychologists in the workplace‐based program, particularly in the way information was shared by psychologists. They also highlighted how the sharing of information with employers impacted COVID‐related stigma.

“It was tough for everyone, Fear of the unknown. After the employer organized debriefing, I thought I would be relieved, but the way those psychologists shared our information, it was disappointing. Once people suspect that you have COVID, they would act somehow (strangely) towards you, so if I shared with the de‐briefer, I sort of expected my information to be confidential because I was at home and no one was at risk on my account” (P4, 22 female).

### 6.3 Experiences of Working Conditions

#### 6.3.1 Safety Protocols and Personal Protective Equipment

Most social workers felt that safety protocols gradually improved as the lockdown was adjusted to other levels because at level 5, safety and protocol were not up to standard due to a lack of sufficient knowledge about COVID‐19.

“I think as time went on, things became better, but in the beginning, it was tough. I remember the center was never going through the decontamination processes until we had to address it with management. When we heard there was a positive case, we demanded that those people must come and decontaminate before we could report to work. No protocols were followed here; sometimes I feel like the government just wants the work done, and workers don't matter. Imagine going back to sit in the center within 3 h of decontamination when it is said that thing they use is not safe” (P1, 22 female)

#### 6.3.2 Use of Masks During Telephonic Counselling

Social workers stated that they had to wear masks for the best part of the day. The only time one was allowed to be seen without a mask was when talking to a client, but put it back on immediately after they hung up.

“Let me tell you, it was hard. Working telephonically and having to wear a mask was not so good. Imagine talking to a client, then you put on a mask after each call, it was not a good moment at all” (P5, 26 female).

### 6.4 Workload and Performance

#### 6.4.1 Work Overload and Its Impact on Performance

As much as the social workers mentioned being overwhelmed, they surprisingly managed to find positivity in the situation. High call rates from GBV victims meant that all social workers met their expectations as far as the quantity targets were concerned. This may have been indicative of the call demand from the general public for GBV services. Some targets were doubled, and some even tripled. At the same time, they expressed how busy and pressurizing it was for them to manage call loads.

“I think we were all compelled to perform at that time, and we were working under pressure, so I will say that we had to work harder than before. So, I think we all excelled then because the caseload was very high” (P1, 52 female).

#### 6.4.2 Workload and Its Impact on Quality of Work

In addition to overwhelming GBV cases and COVID‐19 uncertainties, some participants explained that the workload affected the quality of their overall work. They stated that capturing information on the command center system was affected, given the very high call rates they experienced during this period.

“I was burnt out. I had the highest number of cases compared with other years at the command center. I would receive calls and attend to cases, but sometimes I couldn't populate the system fully. I'd make comments in the box, but not complete the full psychosocial report. So, while services were rendered, the only record was the comment box. If someone searched the system later, they'd find minimal information” (P2, 48 female).

#### 6.4.3 Work–Life Balance

Most social workers expressed that they also expected to do their GBVCC work from home during the COVID‐19 lockdown period, like most employees, but they were unable to do so because the center was expected to run 24/7 regardless. They compared the center with many other call centers that were taking calls from home during the COVID‐19 lockdown period, which allowed them to observe their families while working at home. They spoke of “life without balance”. They found work–life balance was impacted when having to work on the GBV pandemic while the whole country was observing COVID‐19. Some participants mention having missed out on other opportunities outside of work.

“Uhm, there was no balance, it was just work, home, work, home. It's just back and forth and I don't know if there was anything full‐time and then I'd be home watching TV or if you like spending time on the phone you would do that, so I think it was just about the work, nothing else that you would think of because most of the things you'd pause, I remember I had even started, uhm, I think a month or two before COVID, I started wellness practice, but because of the fear and everything, I had to stop, yeah, so it was just about this, the full time employment, I had to focus on that” (P4, 36 female).

### 6.5 Lessons Learned and Recommendations

Social workers expressed that the COVID‐19 pandemic taught them that health and safety at work were paramount. Suggestions were that, in the future, the department should consider having a designated workstation for each social worker instead of sharing workspaces and equipment.

“I think even the tools of trade, you know; I think if it is possible or feasible that every official can own their own workstation, you know, to promote that sense of safety, and also I think it is about the working environment” (P2, 54 female).

Recommendations for suspending shift work have also been suggested by some social workers. They mentioned that they wished that the department had at least shortened the hours to eight instead of 12 h.

“I think there was a year when we started working in the center where supervisors would take mobile phone and calls would be diverted to them around festive season. I think that could work in future, you know, to get tools of trade that can allow us to work from home during such times. Working was a struggle, to be honest, and I, I, I think shifts were supposed to be stopped just for that time, you know, to at least work from 8 to 4 (laughing) or just close the center just like all other departments. Imagine the cubicles, guys, that moment made me sick.” (P8, 42 female).

## 7 Discussion

This study explored the impact of the 2020–2021 COVID‐19 adjusted lockdown on social workers at a gender‐based violence command center in Pretoria, Gauteng. It provides first‐hand accounts of the impact of COVID‐19 and its related lockdown on mental health, coping behaviors, workload, performance, and work–life balance among nine social workers employed at the GBVCC. An overarching theme that emerged from the participants was the impact on their mental health, most notably stress, anxiety, and depression when working during this time, which was often associated with a fear of death. This is consistent with other studies that found that the psychological effects of pandemics can be intense, significant, and long‐lasting, and typically include a variety of psychological symptoms, such as stress, anxiety, and depression [17]. The study conducted in South Africa post‐COVID‐19 lockdown found that the COVID‐19 pandemic increased the stress experienced by frontline healthcare workers and helpline social workers [18]. The findings of this study underscore the urgent need for thorough support networks for healthcare providers, social workers, and all essential personnel engaged in violence against women (VAW) service delivery. This includes mental health services and sufficient protective measures to maintain their well‐being and ability to provide vital services [18].

Our study findings correlate with the existing evidence that all participants have experienced these feelings in one way or another. Although all participants were in the helping profession, they found themselves battling anxiety, highlighting the need for mental health and self‐care for professionals during pandemics [6, 7].

The family as an institution helped participants cope with their daily life experiences. The different levels of socioecological systems, such as individual, family, community, societies, environment, and spiritual dimensions, influence each other and contribute significantly to limiting the impact of isolation and feelings of anxiety during the COVID‐19 lockdown [17]. In this study, the domains of family and friends had a significant influence on the lives of participants. During the pandemic, participants reported that they used their available support systems to survive ongoing trauma. These sources of support exist across different levels of social ecology, from friends, family, and colleagues. This is in keeping with the socioecological framework, which posits that individual lives and behaviors affect and are affected by multiple levels of influence within the individual and in their environments [19].

These findings show that, as much as the GBV workload was heavy, social workers managed to meet their expected performance indicators in a short period of time compared with other years. However, the pandemic negatively affected their work–life balance. This highlights the resilience and commitment of social workers during the pandemic, despite the personal toll on their well‐being.

One limitation of this study was its exclusive focus on social workers, thereby excluding other professionals who were integral to the center's operations during the lockdown period. Future research could benefit from including a broader range of professionals involved in the center's operations, such as healthcare providers, administrative staff, and support personnel. This more comprehensive approach would provide a holistic view of the challenges faced and strategies employed during the lockdown period, potentially revealing interdisciplinary insights and collaborative solutions that were not within the scope of the current study.

Based on the collective views of social workers who participated in the study, there is evidence that there is greater caution than usual in South Africa regarding health and hygiene due to the COVID‐19 pandemic [20]. Social workers’ ideas on future preparedness concur with studies conducted in other countries during the same period. One study on mental well‐being during the COVID‐19 lockdown found that mental health imbalances were prevalent during the hard lockdown period, and most of the participants relied on their families to cope [17].

Further suggestions by social workers included the redirection of services by diverting the calls to allow social workers to work from home during the pandemic. This step requires the department to invest in tools of trade that will make it possible to work from home, should the situation be dire.

## 8 Conclusion and Recommendations

The study concludes that poor mental health and related problems are a large and complex phenomenon in the workplace, particularly in the face of pandemics. Mental health problems in employees also place a burden on society as a whole, health systems, employers, those affected, and their families [21]. It is therefore vital to prepare for future pandemics by developing programs in the workplace to address mental health issues regularly. Social workers’ input should be taken into consideration when developing standard operating procedures (SOP), so that their recommendations can be operationalized both routinely and during pandemic situations.

Several strategies have been implemented to support the psychological well‐being of healthcare workers, especially during the COVID‐19 pandemic. These strategies aim to mitigate stress, burnout, and other mental health challenges, such as fear of death and anxiety, faced by healthcare professionals [11]. One key approach has been the establishment of psychological support hotlines, which provide accessible emotional support for healthcare workers facing stress and anxiety [22]. Social workers have recommended these interventions at a primary level for better working conditions and for future mitigation.

## Author Contributions

Ms. Qebo Nkosi participated in the study from the inception, design, data collection, data analysis, and drafting of the manuscript. Dr. Leane Ramsoomar‐Hariparsaad supervised the research process and critically reviewed the manuscript. All authors approved the final version of the manuscript.

## Conflicts of Interest

The authors declare no conflicts of interest.

## Data Availability

The data supporting the findings of this study are available from the corresponding author upon reasonable request.
